# A four-year-old neglected traumatic bipolar clavicular dislocation: a case report

**DOI:** 10.1016/j.xrrt.2021.03.007

**Published:** 2021-04-20

**Authors:** Mohamed Kamal Mesregah, Bahaa Zakarya Hasan

**Affiliations:** Department of Orthopaedic Surgery, Faculty of Medicine, Menoufia University, Shebin El-Kom, Menoufia, Egypt

**Keywords:** Bipolar clavicular dislocation, floating clavicle, chronic clavicle dislocation, sternoclavicular joint, acromioclavicular joint, total claviculectomy

Bipolar clavicular dislocation, simultaneous the dislocations of the sternoclavicular (SC) and acromioclavicular (AC) joints, is a rare injury, most commonly caused by major trauma.^9^^,^^12^ This condition has also been referred to as bifocal clavicular dislocation, floating clavicle, periarticular clavicle dislocation, and panclavicular dislocation.^2^

Porral^11^ described the first case of bipolar clavicular dislocation in 1831, and since then, a scarce number of reports have been published. Therefore, there is limited knowledge of the diagnosis, the optimal treatment, and the prognosis, especially in chronic cases.

The prominent deformity and instability associated with this type of injury may account for the need to treat it surgically, especially in high-demand young patients.^8^^,^^16^ Different surgical methods have been used and achieved good results, with most cases diagnosed early.^1^^,^^9^^,^^14^^,^^15^ Throughout the literature, Argintar et al^2^ reported the only case of traumatic bipolar clavicular dislocation treated by complete claviculectomy.

In this report, we sought to present a patient with a four-year chronic neglected bipolar clavicular dislocation treated by total claviculectomy.

## Case report

A 26-year-old male manual worker sustained a high-velocity motor vehicle accident and fell on the ground with the focus of impact centered on his left shoulder's anterolateral aspect. He was brought to a nearby emergency hospital, where he had undergone the necessary investigations to exclude any serious issues related to the trauma.

The main complaint was pain and swelling over the left shoulder girdle. On examination, there was tenderness, swelling, and deformity with a painful range of motion. Chest X-ray showed SC joint dislocation for which he had undergone closed reduction under sedation, and a figure of eight was applied. However, he revisited after two weeks and reported feeling a protrusion and discomfort at the lateral end of the clavicle. Repeated chest X-ray revealed additional dislocation of the AC joint. Surgical intervention for both SC and AC joint dislocations was discussed with him. However, he refused and insisted on continuing conservative treatment.

Four years later, he presented to our clinic with the complaint of feeling embarrassed and mentally distressed owing to the cosmetic issue and continuous motion of the floating clavicle, which had started to be noticed by his children. This cosmetic issue badly affected his quality of life.

On examination, a noticeable deformity was observed on his left shoulder girdle. The medial end of the clavicle was anterosuperior to the sternum, and the acromion was prominently visible. The clavicle was freely mobile without tenderness.

Examination of the shoulder range of motion revealed a similar range between the left shoulder and the uninjured right shoulder with 90° of abduction, 170° of forward flexion, in addition to symmetric external and internal rotation in both sides. He also could raise both arms overhead and showed no hand impairment. In addition, the muscles' strength was symmetric, and there was normal light touch sensation along all dermatomes.

**Figure 1:**
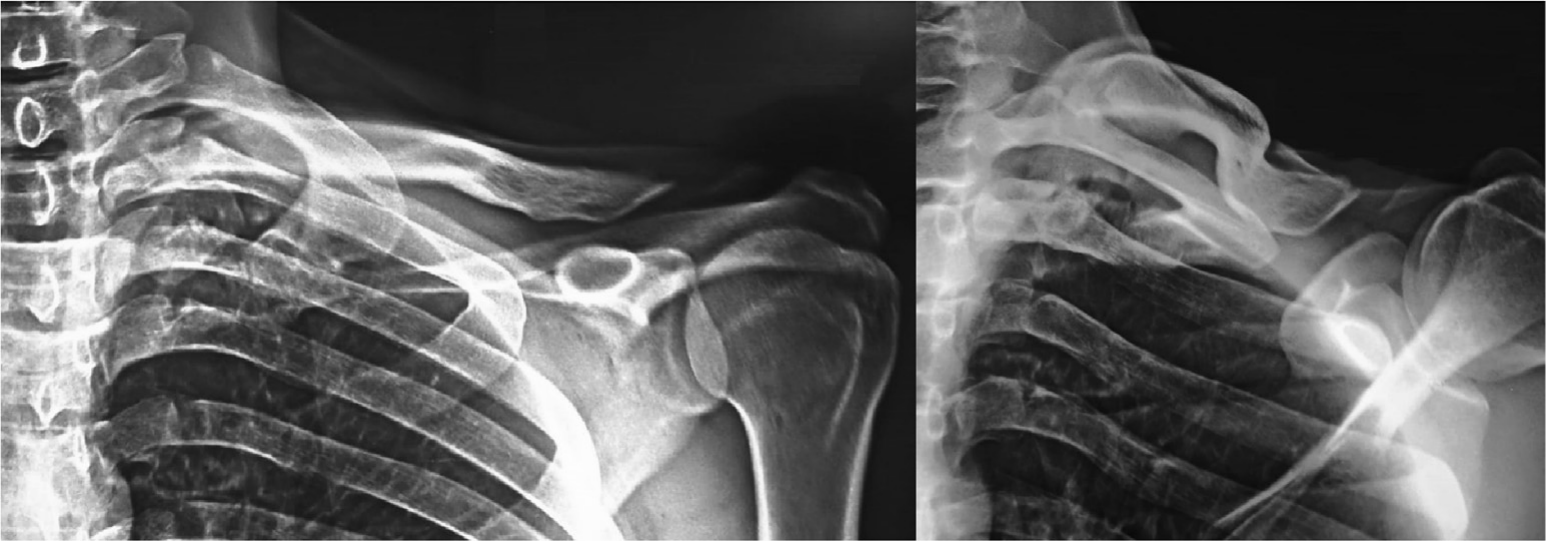
Preoperative radiographs of the shoulder girdle showing bipolar dislocation of the clavicle.

Plain X-rays demonstrated bipolar clavicular dislocation with anterosuperior displacement of the medial end and posteroinferior displacement of the lateral end of the floating clavicle, Figure 1.

The surgical plan of evaluating the SC and AC joints for possible reconstruction or total claviculectomy was discussed with him.

**Figure 2:**
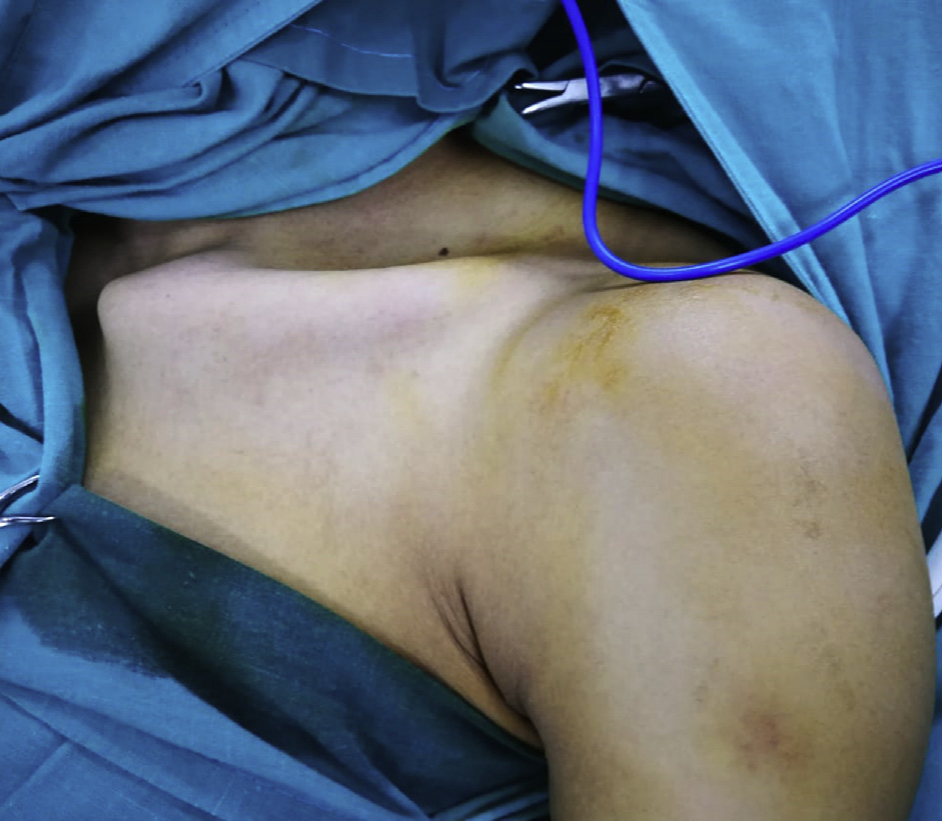
Photograph of the floating clavicle and its cosmetic appearance.

He was placed in a beach-chair position after general anesthesia, with adequate exposure of the whole clavicle and both SC and AC joints, Figure 2. A 10-cm skin incision was performed down to the bone over the prominent clavicle, which was freely mobile with its medial and lateral ends encased in scar tissues. Both ends of the clavicle showed no viable cartilage, which made the joint reconstruction option nonfeasible. Accordingly, total clavicle resection was decided.

**Figure 3:**
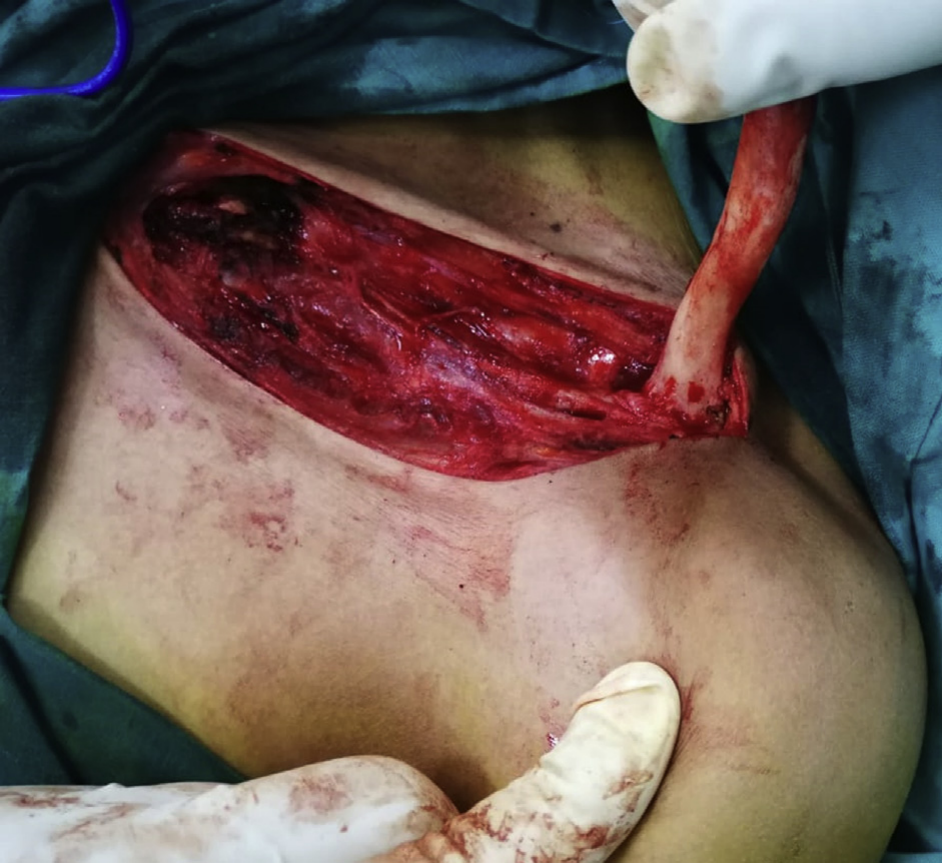
En bloc resection of the clavicle after subperiosteal dissection.

**Figure 4:**
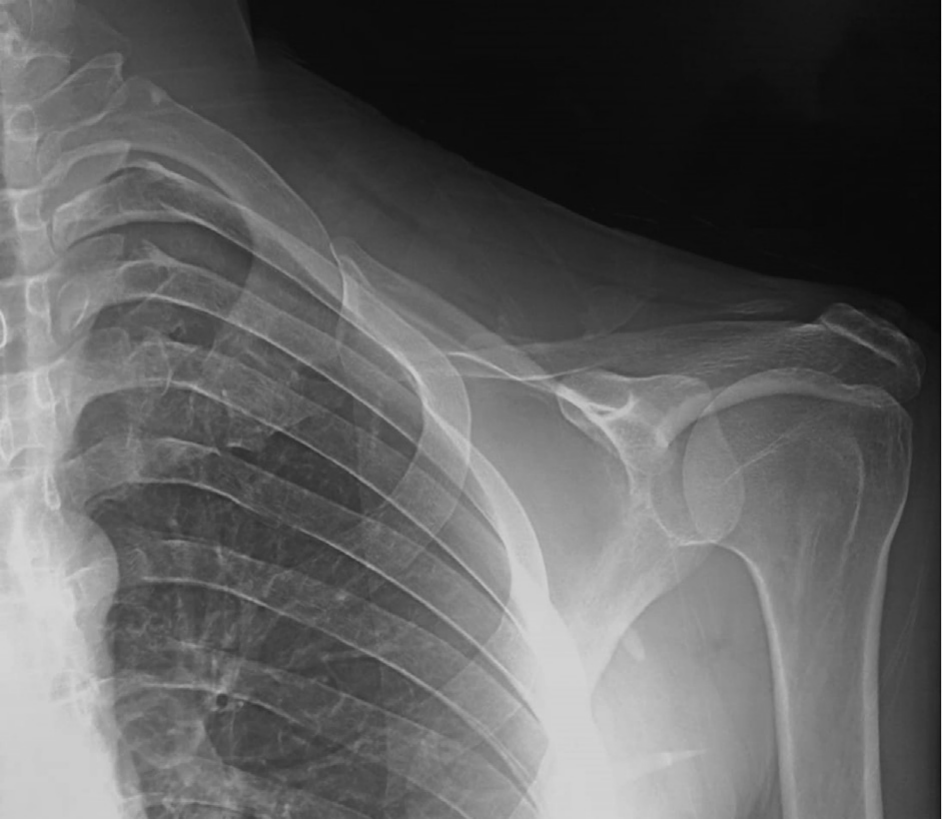
Postoperative radiographs of the shoulder girdle after total claviculectomy.

Meticulous subperiosteal dissection of the clavicle was performed to avoid injuring the pleural cavity and the nearby brachial plexus, and en bloc resection of the clavicle was conducted Figure 3. Muscles were reattached and sutured to the soft tissue along the resected clavicle line. The skin was sutured after inserting a drain that was removed on the following day. Postoperative plain X-rays were obtained Figure 4.

**Figure 5:**
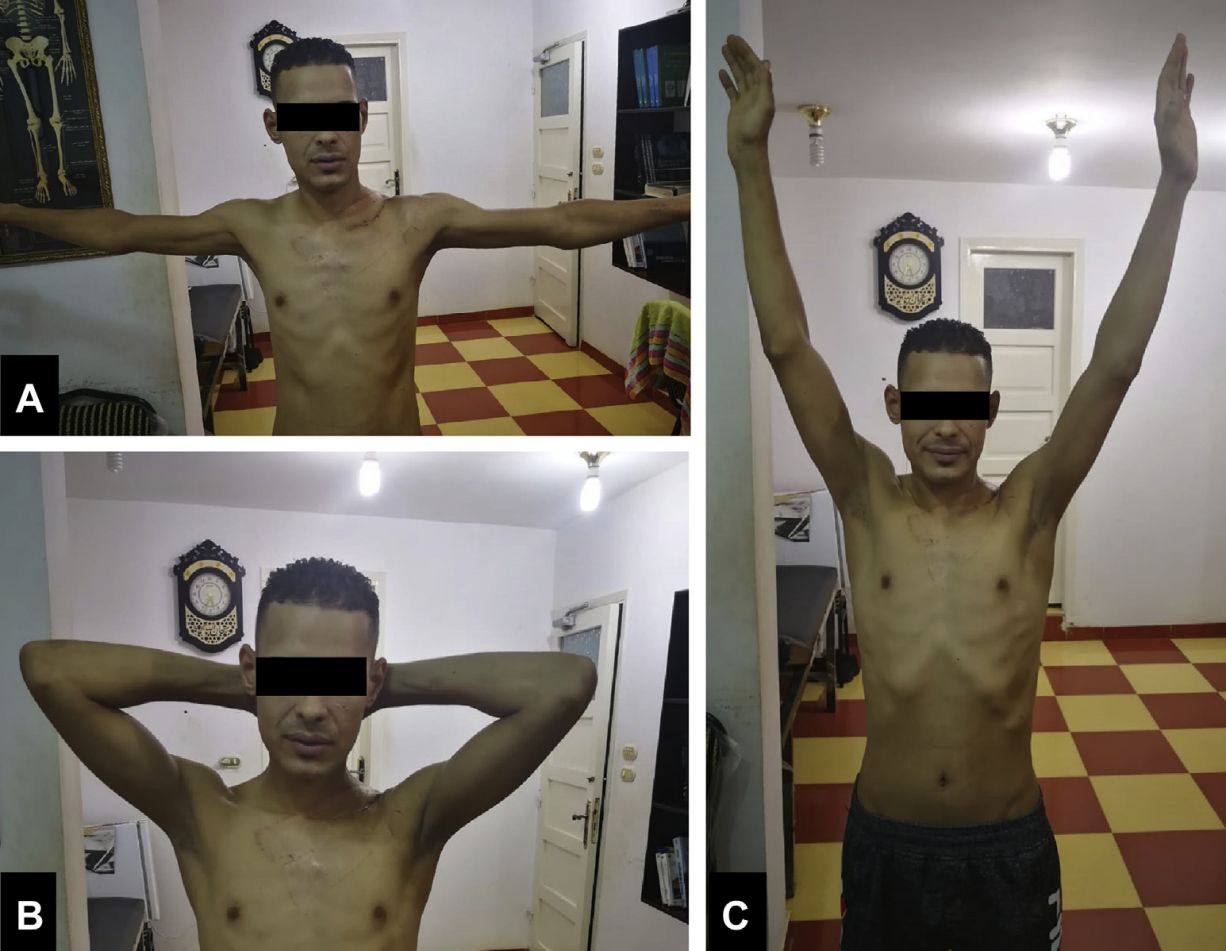
Shoulder range of motion two weeks after total claviculectomy. (**A**) Abduction. (**B**) Hands behind the head to test for the abduction and external rotation. (**C**) Forward flexion.

Active shoulder motion started early at 1 week postoperatively, Figure 5. Sutures were removed after two weeks. On follow-up 1 month postoperatively, the range of motion and muscle strength were full and symmetrical between both sides. He was highly satisfied with the treatment and was able to resume his daily life activities normally. The clinical improvement was maintained at the final follow-up examination at 1 year.

## Discussion

The term bipolar clavicle injury has been used to indicate several types of dislocation or fracture of both ends of the clavicle, without any specific classification system.^8^ True bipolar dislocations have been reported a few times in the literature.

In this study, we presented a patient with chronic bipolar clavicle dislocation that was neglected for four years. The main complaint was the cosmetic issues of the prominent clavicle in front of the left shoulder. After total claviculectomy, he was satisfied and returned to his usual daily activities.

The incidence of the two joint dislocations may be sequential, with a delayed diagnosis of 1 joint dislocation. In our report, the SC joint dislocation was diagnosed first, and the AC joint dislocation was diagnosed several days later. Cook et al^5^ reported a patient who sustained SC joint dislocation, and after several months, an additional diagnosis of AC joint dislocation was made.^5^ However, the patient reported that he had felt a pop and mild pain in his shoulder several days after the initial presentation, yet he did not report this to his doctor.^5^ Eni-Olotu and Hobbs^6^ reported a patient with an initial diagnosis of AC joint dislocation who reported neck pain and feeling of pressure on the trachea after 1 week and was subsequently diagnosed with simultaneous SC joint dislocation. Patients with dislocation of one joint of the clavicle should be followed up sufficiently and be advised to revisit if they felt any shoulder pain or discomfort to diagnose dislocation of the other joint early.

In this report, the dislocation occurred in the anterosuperior and posteroinferior directions in the SC and AC joints, respectively. Argintar et al^2^ reported a patient with 2-year chronic bipolar dislocation of the clavicle with similar directions of dislocations.

Owing to the rarity of bipolar dislocation of the clavicle in the literature, treatment remains controversial.^15^ Some authors reported good results after conservative treatment.^7^^,^^10^ However, Eni-Olotu and Hobbs^6^ reported some shoulder weakness and subsequent lateral clavicle excision after conservative treatment.

Different surgical management options have been used effectively for the treatment of bipolar clavicular dislocation. Schemitsch et al^15^ reported good results after open reduction and internal fixation of the two joints using hook plates. Scapinelli^14^ achieved good outcomes after transarticular fixation using three Kirschner wires (K-wires) for the SC joint and two K-wires with a figure-of-eight tension band for the AC joint. Excellent outcomes were obtained by Arenas et al^1^ using open reduction and internal fixation with two K-wires for the AC joint and closed reduction with percutaneous fixation using two K-wires for the SC joint. Sanders et al^13^ treated four patients with chronic traumatic bipolar clavicular dislocation by surgical stabilization of the AC joint without surgery over the SC joint and reported good results without pain in SC joint. Okano et al^9^ reported two patients treated by different methods, and both achieved good results. One patient had a modified Cadenat's procedure and K-wires fixation for the AC joint and conservative treatment with a figure-eight bandage for the SC joint.^9^ The other patient had open reduction and internal fixation with hook plate for the AC joint and open reduction and soft-tissue reinforcement using braided polyethylene-blended sutures for the AC joint.^9^

In a recent systematic review and meta-analysis, Chen et al^4^ compared the outcomes of clavicular reconstruction and claviculectomy in patients with tumors and other disorders and reported similar clinical results, with faster rehabilitation and a lower risk of further surgery in patients with isolated claviculectomy. Accordingly, total claviculectomy for floating clavicle, which is rarely described in the literature, can be considered for this type of pathology. Attarian^3^ described complete excision of the clavicle for nontraumatic floating clavicle, which occurred eight years after anterior shoulder stabilization for recurrent dislocation of the shoulder and ipsilateral lateral clavicle resection, and reported improvement of most symptoms with some weakness and fatigue. Argintar et al^2^ performed total claviculectomy in a 55-years-old man with a 2-year chronic traumatic floating clavicle, and clinical improvement was achieved in the early recovery period.

The patient reported in this study is the second patient in the literature to undergo total claviculectomy for traumatic floating clavicle after the patient reported in Argintar et al^2^ study. Both patients had chronic bipolar dislocation with the intraoperative finding of nonviable SC and AC joints. Calviculectomy is a safe procedure but may be associated with some complications such as vascular laceration and infection.^4^ We believe that total claviculectomy is a possible treatment option for chronic symptomatic floating clavicle as joint reconstruction is not possible because of the degenerative changes that occur in the joints.

## Conclusion

Bipolar dislocation of the clavicle is a rare condition that may be disabling owing to the associated instability and cosmetic issues. Chronic dislocation may cause degenerative changes and nonviability of the SC and SC joints. Total claviculectomy is a possible treatment option for chronic clavicular dislocation with excellent outcomes and high patient satisfaction.

## Disclaimers

Funding: No funding was disclosed by the authors.

The authors, their immediate families, and any research foundations with which they are affiliated have not received any financial payments or other benefits from any commercial entity related to the subject of this article.

Patient consent: Obtained.
